# Psychometric Properties of the POAGTS: A Tool for Understanding Parents’ Perceptions Regarding Autism Spectrum Disorder Genetic Testing

**DOI:** 10.3390/ijerph18063323

**Published:** 2021-03-23

**Authors:** Shixi Zhao, Wei-Ju Chen, Oi-Man Kwok, Shweta U. Dhar, Tanya N. Eble, Tung-Sung Tseng, Lei-Shih Chen

**Affiliations:** 1 Department of Health, Exercise & Sports Sciences, University of New Mexico, Albuquerque, NM 87131, USA; shixizhao@unm.edu; 2 Department of Psychology, the University of Texas of the Permian Basin, Odessa, TX 79762, USA; chen_w@utpb.edu; 3 Department of Educational Psychology, Texas A&M University, College Station, TX 77843, USA; omkwok@tamu.edu; 4 Department of Molecular and Human Genetics, Baylor College of Medicine, Houston, TX 77030, USA; dhar@bcm.edu (S.U.D.); teble@bcm.edu (T.N.E.); 5 Behavioral and Community Health Sciences Program, Louisiana State University Health Sciences Center School of Public Health, New Orleans, LA 70112, USA; ttseng@lsuhsc.edu; 6 Department of Health and Kinesiology, Texas A&M University, College Station, TX 77843, USA

**Keywords:** Autism, genetic testing, psychometric properties, survey

## Abstract

Due to the increased prevalence of Autism Spectrum Disorder (ASD), more children with ASD may be referred for genetic testing. It is important to develop a tool to help parents consider the benefits and drawbacks of genetic testing for ASD before pursuing genetic testing for children with ASD. We developed the first theory-based survey—Perceptions of ASD Genetic Testing Survey (POAGTS), as a tool to assist healthcare providers to better understand parents’ perceptions and concerns regarding ASD genetic testing. The psychometric properties of POAGTS were first pre-tested and then formally tested with 308 parents of children with ASD who had not decided whether to pursue genetic testing for their children diagnosed with ASD. Findings suggest that the eight scales of the POAGTS were psychometrically sound, and had acceptable data reliability and validity. Additional research with various samples, such as parents of children with ASD who belong to diverse racial/ethnic and socioeconomic groups, is warranted in the future to determine whether the POAGTS is applicable to these particular groups. Condensing and refining this tool to a shorter, more user-friendly version is also recommended for future research.

## 1. Introduction

Autism Spectrum Disorder (ASD) is an umbrella term for a group of neurodevelopmental disabilities [1] with a trend of increasing prevalence in the United States (U.S.) [2]. Studies have shown that genetics is one of the main factors contributing to ASD, as hundreds of genes have been identified to be associated with ASD [3,4,5,6,7]. Currently, genetic testing for ASD is available in clinical practice. Several leading medical organizations, including the American Academy of Pediatrics [8,9], American Academy of Child and Adolescent Psychiatry [10], American Academy of Neurology [11], and American College of Medical Genetics and Genomics [12], have established clinical guidelines and recommendations for offering genetic testing for children diagnosed with ASD. Based on clinical indications, several different genetic tests are available [13,14,15,16]. The first-tier genetic tests include chromosomal microarray analysis and Fragile X testing [12,13,14]. Genetic testing for ASD could be beneficial for both children diagnosed with ASD and their parents. For children with ASD, genetic testing may help identify the etiology of ASD and assist in creating a personalized medical treatment plan. For parents of children with ASD, genetic testing can provide information for parents to make informed reproductive decisions [9,12,17].

When a child is diagnosed with ASD, the parents often experience distress, uncertainty, and negative emotional responses such as shock, anger, guilt, sadness, and anxiety [18,19,20]. As the main caregivers of children with ASD, parents under such psychological and emotional distress need to decide whether they want to pursue genetic testing for their children with ASD in a short amount of time, while dealing with other important tasks (e.g., checking for Medicaid eligibility, verifying health insurance coverage for therapy services, and making appointments with therapists). Moreover, similar to parents of children requiring other genetic tests [21,22,23,24], parents of children with ASD may experience anxiety and distress at every stage of the testing (i.e., before, during, and after testing). To alleviate parents’ anxiety and distress and facilitate better services, communication, and healthcare education, it is important to develop a tool to help healthcare providers better understand parents’ perceptions and barriers of pursuing genetic testing for their children with ASD. This tool can also help parents thoroughly consider the benefits and drawbacks of genetic testing for ASD (i.e., scientific utility). Nevertheless, to the best of our knowledge, there is no such tool that aids in understanding parents’ perceptions and concerns regarding ASD genetic testing currently available and implemented in a clinical setting.

Therefore, to address these critical needs, we developed the first theory-based survey, the Perceptions of ASD Genetic Testing Survey (POAGTS). The development of the POAGTS was guided by an integrated theoretical framework based on three social and behavioral theories: the Health Belief Model (HBM) [25,26], the Theory of Planned Behavior (TPB) [27], and Social Cognitive Theory (SCT) [28]. A theory-based survey can assist healthcare providers to better understand what motivates and discourages parents in the pursuit of genetic testing for their children with ASD. Based on parents’ responses, healthcare providers can provide tailored services to address parents’ concerns and meet their needs. The primary purpose of this study was to examine the psychometric properties (i.e., reliability and validity) of the POAGTS using data obtained from a large sample of parents of children with ASD in the United States.

## 2. Materials and Methods

### 2.1. Theoretical Framework

In previous research on factors associated with genetic testing decision-making [29,30,31,32,33,34,35], three health behavior models/theories have been well represented: HBM [25,26], TPB [27], and SCT [28]. Yet, these three models/theories have often been used and applied separately. Past research has shown that an integrated, multi-theoretical model can capture a comprehensive picture of human health behavior [36,37]. Therefore, we developed a theoretical framework that integrates these three theories/models (i.e., HEB, TPB, and SCT) to understand parental decision-making regarding pursuing genetic testing for their children with ASD.

**Figure 1:**
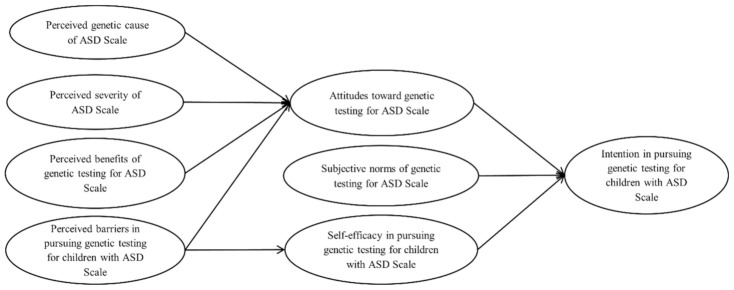
The integrated theoretical framework for the parental intention in pursuing genetic testing for their children with Autism Spectrum Disorder (ASD).

In this integrated theoretical framework (as illustrated in Figure 1), parental intention in pursuing genetic testing for their children with ASD was associated with their attitudes toward genetic testing for ASD, subjective norms of genetic testing for ASD, and self-efficacy in pursuing genetic testing for their children with ASD. Parental attitudes towards genetic testing for ASD were correlated with their perceptions of the genetic causes of ASD, the severity of ASD, the benefits of genetic testing for ASD, and the barriers in pursuing genetic testing for their children with ASD. Furthermore, parental self-efficacy in pursuing genetic testing for their children with ASD was negatively related to the barriers they perceived in pursuing genetic testing for their children with ASD.

### 2.2. Measurement

**Perceived Genetic Cause of ASD Scale.** According to Calsbeek et al. [38], individuals who perceive disease as hereditary tend to have more favorable attitudes toward genetic testing. Thus, we adopted this finding to develop the perceived genetic cause of the ASD scale for this study. This scale included four items examining whether or not parents believe that their children’s ASD was caused by genes. The items were rated using four-point Likert-type scale responses including “strongly disagree”, “disagree”, “agree”, and “strongly agree.”

**Perceived Severity of ASD Scale.** Grounded in the HBM [25,26], the perceived severity of ASD dimension contained six items that assessed parental feelings about potential manifestations or consequences of their children’s ASD condition using a four-point Likert-type scale with the response options of (“strongly disagree”, “disagree”, “agree”, and “strongly agree”).

**Perceived Benefits of Genetic Testing for ASD Scale.** Based on the HBM [25,26], this scale consisted of six, four-point Likert-type rating items including “strongly disagree”, “disagree”, “agree”, and “strongly agree” that examined parental views of the advantages of pursuing genetic testing for their children with ASD.

**Perceived Barriers in Pursuing Genetic Testing for Children with ASD Scale.** Based on the HBM [25,26], nine items using a four-point Likert-type scale (with responses ranging from “strongly disagree” to “strongly agree”) were used to study potential obstacles that might prevent parents from pursuing genetic testing for their children with ASD.

**Attitudes toward Genetic Testing for ASD Scale.** Founded on the TPB [27], we measured both the beliefs and values dimensions of parental attitudes regarding pursuing genetic testing for their children with ASD. Each dimension was measured using five items. The belief subscale used a four-point Likert-type rating of parental agreement levels ranging from “strongly disagree” to “strongly agree”, whereas the value subscale consisted of a four-point Likert-type rating on the levels of importance ranging from “not important at all” to “extremely important.” Each belief item had a corresponding value item, and their respective scores were multiplied to create five composite attitude scores.

**Subjective Norms of Genetic Testing for ASD Scale.** The TPB [27] refers subjective norms to an individual’s perception of what their significant other thinks about him/her in performing a certain behavior (i.e., normative beliefs) as well as his/her motivation to comply with a significant other’s beliefs concerning performing that behavior [27,39]. Each of these two subscales was measured using nine items in this study. In particular, normative beliefs were collected by asking parents their likelihood of compliance if their significant other suggested pursuing genetic testing for their children with ASD. Normative beliefs were rated using a four-point Likert-type scale ranging from “extremely unlikely” to “extremely likely.” Motivation to comply was assessed by asking parents the extent to which they cared about whether or not their significant other thought they should pursue genetic testing for their children with ASD. It was rated on a four-point Likert-type scale ranging from “not at all” to “very much.” Each subjective norm score was calculated by multiplying the score for each individual parent’s normative beliefs by the score for his/her motivation to comply, which created nine subjective norm composite scores.

**Self-Efficacy in Pursuing Genetic Testing for Children with ASD Scale.** Guided by the SCT [28], the six-item self-efficacy dimension of the POAGTS assessed parental confidence in pursuing genetic testing for their children with ASD. Response options ranged from 0 (“I am not confident at all”) to 10 (“I am 100% confident”).

**Intention in Pursuing Genetic Testing for Children with ASD Scale.** As indicated in the TPB [27], intention is an important predictor of one’s motivation to perform a particular behavior. In this study, we examined parental intention to pursue genetic testing for their children with ASD. This six-item scale used a four-point Likert-type rating ranging from “extremely unlikely” to “extremely likely.”

### 2.3. Pretesting the POAGTS

The Institutional Review Board at Texas A&M University approved all the research protocols used in this study. In order to conduct initial testing of the POAGTS, we adopted the procedures recommended by Dillman et al. [40] and DeVellis [41] in three phases.

In phase I, five aspects of the survey were evaluated by a panel of seven experts who reviewed the preliminary version of the POAGTS that included a statistician, an ASD behavior analyst, a special education expert, a geneticist, a genetic counselor, and two social behavior specialists. Specifically, they examined: (1) whether the survey items were applicable to parents of children with ASD, (2) whether the survey items assessed the constructs of interest, (3) if any survey items were redundant or should be eliminated, (4) if any additional items should be added to the survey, and (5) whether the overall design of the survey could be improved. Only a few changes in content and format were made based on these experts’ suggestions.

In phase II, we conducted seven cognitive interviews and five retrospective interviews with a convenient sample of parents of children with ASD in East-Central Texas to examine how the parents of children with ASD perceive, process, comprehend, interpret, and respond to the POAGTS questions/items [40]. Based on the participants’ comments and suggestions, we reworded and reformatted six items in questions associated with the constructs measured in this study. The revision was checked and approved by these experts to ensure the content validity of POAGTS.

Phase III involved administering the POAGTS to a small group of participants, which allowed us to evaluate the survey, study the procedure, and identify any potential problems or difficulties that might arise using the survey with a larger sample [40]. This phase also helped identify patterns of missing data, examine the scales for their capacity to measure the intended theoretically based constructs, and determine whether any particular survey item should be eliminated in formal testing. We invited 300 parents of children with ASD in ASD clinics and organizations in East-Central Texas to participate in the study, of whom 52 completed the pre-test POAGTS. After examining the descriptive statistics, as well as the results of reliability, exploratory factor analysis (EFA), and confirmatory factor analysis (CFA), we revised the wording of a few of the survey items for clarity.

### 2.4. Formal Testing

#### 2.4.1. Data Collection

Biological parents of children diagnosed with ASD were recruited from a national ASD research registry, the Interactive Autism Network (IAN; http://www.iancommunity.org (accessed on 25 July 2020)). The ASD diagnosis of children in the IAN research registry has been clinically validated and confirmed through the examination of medical records [42,43]. Prospective participants (N = 4673) randomly chosen from the IAN research registry were invited to complete the POAGTS via one emailed invitation and three reminder emails. Qualtrics (http://www.qualtrics.com (accessed on 25 July 2020)), a web-based survey platform, was used to host the POAGTS. A $10 electronic gift card was sent to each participant who completed the POAGTS.

#### 2.4.2. Participants

The sample of the formal test consisted of 500 biological parents of children with ASD (response rate = 10.70%). The formal testing (*n* = 500) and pilot testing (*n* = 52) samples were combined for data analysis. This is because only a few minor wording and format changes were made to the POAGTS used in the formal testing compared to the pilot testing version. Additionally, the data collection and analysis procedures for the formal testing were aligned with the pilot testing procedures. No statistically significant differences in any scales or demographic characteristics were found between the pilot and formal testing groups. The combined sample consisted of 552 biological parents of children with ASD, of which 244 parents were excluded from the final data analysis because (1) their children had already undergone genetic testing for ASD, (2) they did not indicate whether they had pursued genetic testing for ASD, and (3) they had declined genetic testing for ASD. The final sample size, therefore, consisted of 308 biological parents of children with ASD who had not decided whether to pursue genetic testing for their children diagnosed with ASD.

The average age of the parents in this study was 44.7 years (Standard deviation [SD] = 7.9 years), and most were mothers (91.9%) and White/Caucasian (86.4%). Slightly over one-fourth of the parents (26.0%) reported having some college or technical school education, and more than two-thirds of participants reported that they were either college graduates (42.2%), or had completed advanced graduate degrees (26.0%). Approximately two-thirds (61.4%) of participants were employed, and more than half (56.8%) of the parents reported that their annual household income was more than $75,000.

#### 2.4.3. Statistical Analyses

Using Statistical Package for the Social Sciences (SPSS) 22.0, we examined the data distribution, descriptive statistics (means, standard deviations, frequencies, and percentages), internal consistencies, and underlying factors of the POAGTS. Internal consistencies (i.e., reliabilities) were evaluated using Cronbach’s alpha (α) coefficients, values of 0.7 or above were considered adequate [44,45]. Underlying factors of each scale were examined by EFA using the oblique promax rotation method [46]. Only factors with eigenvalues larger than 1.25 were retained [47]. Factor loadings were considered as moderately high if they were larger than 0.30, and as high if they were over 0.60 [48]. Factors with fewer than three items identified from EFA were dropped from further analysis. Furthermore, using Mplus 8.0, we conducted CFA using the maximum likelihood estimation method with a robust estimate [49] to check construct validity. Structural equation modeling (SEM) was also performed to evaluate relationships between the latent constructs proposed in the theoretical framework. CFA and SEM model fit statistics included chi-square (χ^2^), root mean square error of approximation (RMSEA), comparative fit index (CFI), and standardized root mean square residual (SRMR). A model with a CFI level above 0.90, an RMSEA value less than 0.10, and an SRMR less than 0.08 was considered to be a “good fit” [50,51].

## 3. Results

### 3.1. Reliability

Table 1 details the means, standard deviations, corrected item total correlations, and Cronbach’s α for each scale of the POAGTS and its corresponding items. The Cronbach’s α coefficients of the scales ranged from 0.73 (Perceived Barriers in Pursuing Genetic Testing for Children with ASD Scale) to 0.90 (Intention in Pursuing Genetic Testing for Children with ASD Scale), suggesting adequate internal consistencies (reliabilities) for the scales.

### 3.2. Exploratory Factor Analysis (EFA)

A Kaiser–Meyer–Olkin (KMO) test was conducted to determine the appropriateness of EFA for each scale of the POAGTS. The KMO indices of the POAGTS scales ranged from 0.66 to 0.88. As all the KMO indices exceeded the 0.50 criterion [45,52], the data for all scales were suitable for EFA analysis. Moreover, Bartlett’s test of sphericity was carried out to determine whether the items within each scale were correlated. All scales were found to be statistically significant (*p* < 0.001), indicating that item correlation existed in each scale. Table 2 provides the items and factor loadings for the POAGTS scales, which are summarized below:

**Perceived Genetic Cause of ASD Scale.** This scale consisted of four items that all loaded onto a single factor and accounted for 64.80% of the total variance. Factor loadings were high and ranged from 0.70 to 0.85.

**Perceived Severity of ASD Scale.** All six items on the perceived severity of ASD scale loaded positively onto a single factor, and accounted for 44.43% of the total variance. The factor loading of the six items ranged from 0.53 to 0.80.

**Perceived Benefits of Genetic Testing for ASD Scale.** The six items presented in this scale loaded onto one factor, which accounted for 46.14% of the total variance. Factor loadings ranged from 0.39 to 0.83.

**Perceived Barriers in Pursuing Genetic Testing for Children with ASD Scale.** Two factors emerged from these nine items and accounted for a combined 47.29% of the total variance. Six of the nine items (i.e., item 4—*Taking my child(ren) with ASD to undergo ASD genetic testing contradicts with my religious or cultural beliefs*; item 5—*ASD genetic testing does more harm than good*; item 6—*ASD genetic testing can cause family conflicts*; item 7—*The procedure of undergoing ASD genetic testing is uncomfortable for my child (i.e., drawing blood)*; item 8—*The results of ASD genetic testing can cause public discrimination against my child(ren) with ASD; and* item 9—*The results of ASD genetic testing can put the health insurance status of my child(ren) with ASD in jeopardy*) positively loaded onto the first barriers factor with coefficients of 0.57 or higher. This factor (i.e., subscale) was interpreted as perceived harms caused by ASD genetic testing and accounted for 31.17% of the total variance. Two items (item 2—*ASD genetic testing cannot improve the current situation of my child(ren) diagnosed with ASD*; and item 3—*My child(ren) has/have already been diagnosed with ASD, so there’s no need to undergo this testing*) loaded onto a second barriers factor (i.e., subscale) that accounted for 16.13% of the total variance, which indicated that parental perception was that ASD genetic testing was unnecessary (i.e., “perceived ASD genetic testing as unnecessary”). The factor loadings for these two items were 0.93 and 0.81. This second barrier factor was dropped from further analysis because it had fewer than three items. The remaining item (item 1—*ASD is not caused by genes*), did not load to any factor and was also dropped from the subsequent data analyses.

**Attitudes toward Genetic Testing for ASD Scale.** The five items on the attitudes scale loaded onto a single factor, accounting for 77.12% of the total variance. The coefficient of each item was above 0.83.

**Subjective Norms of Genetic Testing for ASD Scale.** A single factor emerged from the nine items on the subjective norm scale, accounting for 55.37% of the total variance. All items displayed statistically significant coefficients of 0.60 or higher.

**Self-Efficacy in Pursuing Genetic Testing for Children with ASD Scale.** The six items presented in the self-efficacy scale merged into one factor, accounting for 56.06% of the total variance. Coefficients of the items ranged from 0.62 to 0.87, indicating high factor loadings.

**Intention in Pursuing Genetic Testing for Children with ASD Scale.** Six items in the intention scale loaded onto one factor and accounted for 67.19% of the total variance. The factor loadings ranged from 0.66 to 0.91.

### 3.3. Confirmatory Factor Analysis (CFA)

**Figure 2:**
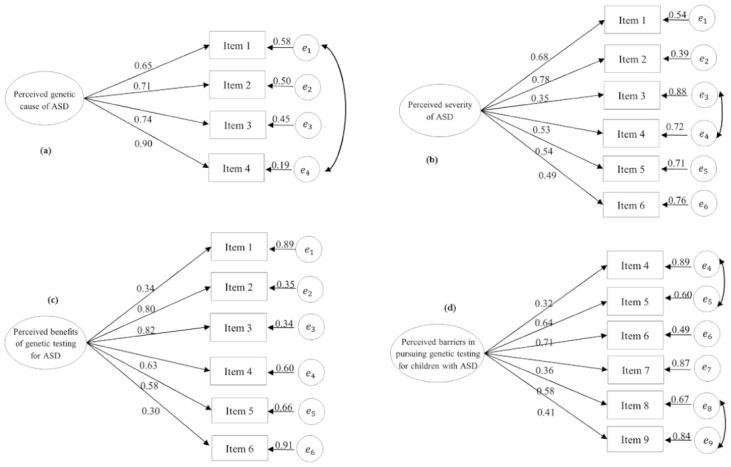

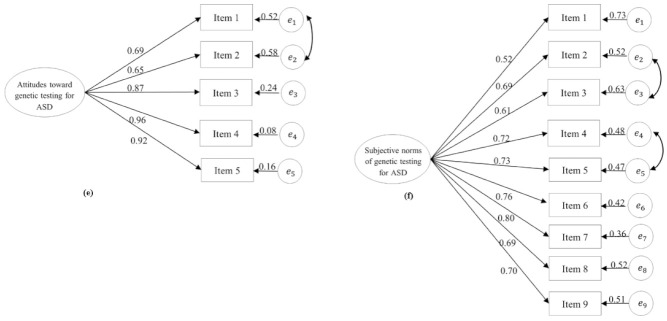

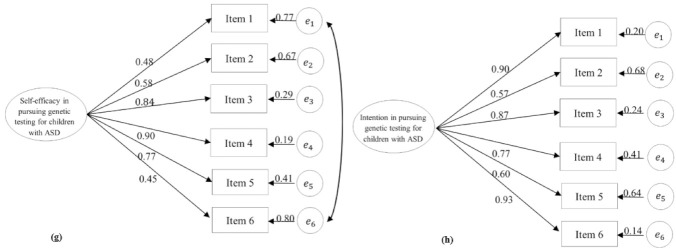
The confirmatory factor analysis (CFA) model for each scale of the POAGTS. (**a**) CFA model for Perceived Genetic Cause of ASD Scale; (**b**) CFA model for Perceived Severity of ASD Scale; (**c**) CFA model for Perceived Benefits of Genetic Testing for ASD Scale; (**d**) CFA model for Perceived Barriers in Pursuing Genetic Testing for Children with ASD Scale; (**e**) CFA model for Attitudes toward Genetic Testing for ASD Scale; (**f**) CFA model for Subjective Norms of Genetic Testing for ASD Scale; (**g**) CFA model for Self-efficacy in Pursuing Genetic Testing for Children with ASD Scale; (**h**) CFA model for Intention in Pursuing Genetic Testing for Children with ASD Scale. Note. The coefficients presented in each scale are standardized coefficients and all of them are statistically significant (*p* < 0.05).

Two scales, including the *Perceived Benefits of Genetic Testing for ASD* and *Intention in Pursuing Genetic Testing for Children with ASD*, yielded a good model fit, suggesting good construct validity. The remaining scales (i.e., *Perceived Genetic Cause of ASD Scale, Perceived Severity of ASD, Perceived Barriers in Pursuing Genetic Testing for Children with ASD Scale, Attitudes toward Genetic Testing for ASD Scale, Subjective Norms of Genetic Testing for ASD Scale, and Self-efficacy in Pursuing Genetic Testing for Children with ASD Scale*) were refined based on the modification indices, and on whether the context of the items with correlated residuals was similar. The revised CFA results indicated that those scales fit the structural models adequately. The specific structure of the CFA model for each scale is illustrated in Figure 2. The detailed CFA findings for each scale are presented below.

**Perceived Genetic Cause of ASD Scale**. The initial four items in the *Perceived Genetic Cause of ASD* Scale yielded only a mediocre model fit (χ^2^ = 23.37, df = 2, *p* < 0.001, RMSEA = 0.19, CFI = 0.96, and SRMR = 0.03). The modification indexes indicated that correlations existed between the error/residual terms of item 1 (“*ASD has a genetic factor*”) and item 4 (“*ASD is an inherited disorder*”). We assumed that these correlations reflected non-random measurement errors due to the overlap of the meaning of the items. The finalized model fit improved (χ^2^ = 1.48, df = 1, *p* = 0.22. RMSEA = 0.04, CFI = 1.00, and SRMR = 0.01) with the added correction between item 1 and item 4. The standardized factor loadings were statically significant and ranged from 0.65 to 0.90.

**Perceived Severity of ASD Scale.** The preliminary CFA findings of this six-item scale yielded only a mediocre model fit (χ^2^ = 51.79, df = 9, *p* < 0.001, RMSEA = 0.12, CFI = 0.89, and SRMR = 0.06). Based on the modification indexes, correlations between the error terms of item 3 (“*The public discriminates against individuals with ASD*”) and item 4 (“*Individuals with ASD have fewer job opportunities*”) were added. These correlations suggested that these items shared some similarities. This revised six-item scale model fit the specified single factor model well (χ^2^ = 24.69, df = 8, *p* < 0.01, RMSEA = 0.08, CFI = 0.95, and SRMR = 0.04). The standardized factor loadings were statistically significant, ranging between 0.35 and 0.78.

**Perceived Benefits of Genetic Testing for ASD Scale.** The six-item model of the *Perceived Benefits of Genetic Testing for ASD Scale* fit the one-factor model adequately (χ^2^ = 20.91, df = 9, *p* = 0.013, RMSEA = 0.07, CFI = 0.97, and SRMR = 0.04). The standardized factor loadings were statistically significant, ranging between 0.30 and 0.82.

**Perceived Barriers in Pursuing Genetic Testing for Children with ASD Scale.** Based on the EFA results, three items were dropped from the CFA analysis. The remaining six items in such subscale were not compatible with the one-factor model (χ^2^ = 123.90, df = 9, *p* < 0.001, RMSEA = 0.20, CFI = 0.73, and SRMR = 0.09). Two measurement error covariances were added in the specified model according to the modification index: item 4 (“*Taking my child(ren) with ASD to undergo ASD genetic testing contradicts with my religious or cultural beliefs.*”) was correlated with item 5 (“*ASD genetic testing does more harm than good*”), and item 8 (“*the results of ASD genetic testing can cause public discrimination against my child(ren) with ASD*”) was correlated with item 9 (“*the results of ASD genetic testing can put the health insurance status of my child(ren) with ASD in jeopardy*”). Both item 4 and item 5 indicated negative thoughts regarding genetic testing for ASD, and both items 8 and 9 represented potential negative consequences associated with genetic discrimination. The added correlation between these four items improved the model fit (χ^2^ = 11.00, df = 7, *p* = 0.14, RMSEA = 0.04, CFI = 0.99, and SRMR = 0.03). All the standardized factor loadings were statistically significant, ranging from 0.32 to 0.71.

**Attitudes toward Genetic Testing for ASD Scale.** The initial attitudes scale did not have a good fit (χ^2^ = 290.63, df = 5, *p* < 0.001, RMSEA = 0.43, CFI = 0.81, and SRMR = 0.09). Based on the modification indexes, one measurement error covariance needed to be added to the model. In particular, the measurement error of item 1 (“*taking a child with ASD to undergo ASD genetic testing is a good thing*”) was correlated with the measurement error of item 2 (“*taking a child with ASD to undergo ASD genetic testing is beneficial*”). Given that both items 1 and 2 shared a close context, we followed the suggestions of the modification indexes to add those residual correlations. The revised factor demonstrated a good model fit (χ^2^ = 11.38, df = 3, *p* = 0.02, RMSEA = 0.08, CFI = 1.00, and SRMR = 0.02). This construct also had statistically significant standardized factor loadings ranging from 0.65 to 0.96.

**Subjective Norms of Genetic Testing for ASD Scale.** The original subject norms scale did not yield a good model fit (χ^2^ = 310.86, df = 27, *p* < 0.001, RMSEA = 0.19, CFI = 0.82, and SRMR = 0.07). The modification indices suggested that two correlations needed to be added for the error terms. The first error term correlation was item 2 (“*my family members on my side*”) and item 3 (“*my family members on my spouse’s side*”). The second error term correlation was item 4 (“*physician*”) and item 5 (“*healthcare professionals other than physicians, such as nurses, social workers, occupational/physical/speech therapists, psychologists*”). Clearly, items 2 and 3 reflected the subjective norms of family members of both sides, and items 4 and 5 implied the subjective norms of healthcare professionals. Thus, the correlations among these residuals were considered to be non-random. The revised model had a good fit (χ^2^ = 73.61, df = 25, *p* < 0.001, RMSEA = 0.08, CFI = 0.95, and SRMR is 0.05). This construct had statistically significant standardized factor loadings ranging from 0.52 to 0.80.

**Self-Efficacy in Pursuing Genetic Testing for Children with ASD Scale.** At the beginning, this six-item self-efficacy scale only had a mediocre model fit (χ^2^ = 64.17, df = 9, *p* < 0.001, RMSEA = 0.14, CFI = 0.93, and SRMR = 0.06). The modification indices suggested that item 1 (“*How confident are you in finding a time to take your child(ren) with ASD for ASD genetic testing?*”) was correlated with item 6 (“*How confident are you that your family members will support you in taking your child(ren) with ASD to undergo genetic testing for ASD?*”). These measurement error term correlations suggested that these items shared commonalities in terms of wording and content. The added measurement error covariances between items 1 and 6 improved the model fit (χ^2^ = 24.83, df = 8, *p* < 0.01, RMSEA = 0.08, CFI = 0.98, and SRMR = 0.03). The standardized factor loadings ranged from 0.45 to 0.90, and all of them were statistically significant.

**Intention in Pursuing Genetic Testing for Children with ASD Scale.** The six-item scale indicated a good model fit (χ^2^ = 17.62, df = 9, *p* = 0.04, RMSEA = 0.06, CFI = 0.99, and SRMR = 0.02). The standardized factor loadings were statistically significant with a range from 0.60 to 0.93.

### 3.4. Structural Equation Modeling (SEM)

SEM was performed to evaluate the relationships among the latent constructs proposed in the theoretical framework. The initial model fit indices (RMSEA = 0.06, CFI = 0.87, and SRMR = 0.09) suggested a mediocre fit. Based on the modification indices and theoretical justification, two paths (i.e., subjective norms of genetic testing for ASD → self-efficacy in pursuing genetic testing for children with ASD; and subjective norms of genetic testing for ASD → attitudes toward genetic testing) were added to the SEM model. The revised model fit improved (RMSEA = 0.05, CFI = 0.90, and SRMR = 0.07) and suggested an adequate model fit. Parents’ attitudes toward genetic testing for ASD (β = 0.26, *p* < 0.001), subjective norms of genetic testing for ASD (β = 0.16, *p* < 0.001), and self-efficacy in pursuing genetic testing for children with ASD (β = 0.57, *p* < 0.001) exhibited positive and statistically significant relationships with parental intention in pursuing genetic testing for children with ASD. There was a statistically significant relationship between parental attitudes toward genetic testing for ASD and the perceived severity of ASD (β = 0.20, *p* < 0.01), perceived benefits of genetic testing for ASD (β = 0.24, *p* < 0.01), and subjective norms of genetic testing for ASD (β = 0.32, *p* < 0.001) in a positive way. However, parental attitudes toward genetic testing for ASD was negatively associated with perceived barriers in having their children with ASD tested (β = −0.30, *p* < 0.01). Yet perceived genetic cause of ASD showed a non-statistically significant correlation with parental attitudes toward genetic testing for ASD (β = −0.02, *p* = 0.81). Parents’ self-efficacy in pursuing genetic testing for children with ASD was positively related with their subjective norms of genetic testing for ASD (β = 0.32, *p* < 0.001), yet negatively associated with their perceived barriers in pursuing genetic testing for children with ASD (β = −0.46, *p* < 0.001).

## 4. Discussion

Due to the increased prevalence of ASD in the U.S. [2], it is anticipated that there will be an increasing trend for children diagnosed with ASD to undergo genetic testing. As parents are often the main decision-makers for pursuing genetic testing for children with ASD, it is important to develop a tool that benefits both parents and healthcare providers in clinical practice. To the best of our knowledge, this study is the first to develop a genetic testing tool for ASD (POAGTS) that has been tested with a large sample of biological parents of children with ASD. Our findings suggested that the POAGTS demonstrated adequate reliability as indicated by high α coefficients, inter-item correlations, and correlation coefficients. Based on the results of EFA, CFA, and SEM, the POAGTS also had acceptable validity. Thus, the POAGTS has the potential to be used as a tool in clinical practice.

It is worth noting that although factor 2 (perceived ASD genetic testing as unnecessary) of the *Perceived Barriers in Pursuing Genetic Testing for Children with ASD Scale* was dropped from CFA analysis due to only two items being identified through EFA analysis, it is still an important sub-domain. This is consistent with previous studies [53,54,55,56] that showed that parents of children with ASD were concerned about the necessity, practical benefits, and usefulness of genetic testing for children with ASD. Future research should develop additional items to measure this dimension, which can assist in better understanding parental concern regarding the necessity of genetic testing for their children with ASD. Furthermore, item 1 (“*ASD is not caused by genes*”) was dropped from the *Perceived Barriers in Pursuing Genetic Testing for Children with ASD Scale* because it did not significantly load onto any of the factors identified in the EFA analysis (i.e., perceived harm caused by ASD genetic testing and perceived ASD genetic testing as unnecessary). This may suggest that the cause of ASD as perceived by parents was not as significant to them as their views regarding potential harm and the need for genetic testing of their children with ASD.

The initial CFA model fit for the six latent constructs (i.e., *Perceived Genetic Cause of ASD Scale, Perceived Severity of ASD Scale, Perceived Barriers in Pursuing Genetic Testing for Children with ASD, Attitudes toward Genetic Testing for Children with ASD, Subjective Norms of Genetic Testing for ASD, and Self-efficacy in Pursuing Genetic Testing for Children with ASD*) was mediocre. In order to improve the model fit of these six latent constructs, one or more correlated residuals/errors were added in each of the CFA models. The added measurement error correlations were based on modification indices, and the extent of the overlap and similarities of the wording and content of the correlated items. Although the correlated residuals improved the model fit, the correlated residuals might also suggest important information, such as the overlap and/or similarities of the wording and content of the correlated items/indicators [57,58], unaccounted for or unobserved factors beyond the hypothesized model (i.e., some of the shared variance in the indicators is due to the identified factor, whereas some of the variance is due to other factors that were not originally hypothesized) [58], the plausible results of the positively and negatively worded items, or the potential existence of higher-order constructs [58,59].

The SEM analysis confirmed the relationship among the latent constructs (i.e., scales) proposed in the theoretical framework, except for the relationship between the perceived genetic cause of ASD scale, and the parents’ attitudes toward genetic testing for ASD scale. This statistically non-significant relationship was at odds with previous literature [38], which suggested that individuals who perceived their own illness as an inherited disease would have more positive attitudes toward genetic testing. This discrepancy might be because parental views of the seriousness of ASD, the potential benefits of genetic testing for ASD, and the obstacles to pursuing genetic testing for their children with ASD outweighed their perceptions of the genetic causes of ASD. Another possible explanation could be that parental attitudes toward genetic testing for their own diseases and genetic testing for their children with ASD are different. Future research is needed to examine the relationship between parental beliefs of the genetic causes of ASD and their attitudes towards genetic testing for their children with ASD.

Researchers should be aware of limitations when using the POAGTS. We tested the survey with participants recruited through the IAN online registry, most of whom were White/Caucasian and had high income and educational levels. Although these characteristics are common in parents of children with ASD [60,61], it is important to further test the psychometric properties of the POAGTS with parents who have limited access to the Internet, as well as those with diverse racial and ethnic minority backgrounds and/or low socioeconomic status in the future. Second, the response rate of this study was relatively low, which may limit the generalizability of this study. Nevertheless, this low response rate is consistent with other studies that have used the IAN database [60,62,63]. Additionally, the demographic characteristics of participants in this study were comparable to other surveys conducted by the IAN [64]. Furthermore, the POAGTS has 65 items, which may overwhelm parents during the time of their children’s ASD diagnosis. To lessen the burden on parents, future research is recommended to condense and refine this tool to a shorter, more user-friendly version.

## 5. Conclusions

The POAGTS was developed based on a multi-theoretical model integrating the HBM [25,26], TPB [27], and SCT [28]. To the best of our knowledge, it is the first theory-based tool for healthcare providers to understand parents’ perceptions regarding genetic testing for their children with ASD. Although the POAGTS showed adequate validity and reliability, further examination of the psychometric properties of this survey in diverse populations via factorial invariance [65] is recommended. Specifically, testing the POAGTS with parents of children with ASD from diverse racial, ethnic and socioeconomic groups is needed to determine whether the POAGTS is applicable to diverse populations. Additionally, work to condense and refine the POAGTS to a shorter version is also recommended to make this tool more user-friendly in the future.

## Figures and Tables

**Table 1:** Means, standard deviations, item-total correlations, and Cronbach’s alpha (α) coefficients for scales within the POAGTS.

Item	Mean	SD	Item Total *r*
Perceived Genetic Cause of ASD Scale (4 items)			
Response Scale: (1) Strongly Disagree, (2) Disagree, (3) Agree, (4) Strongly Agree			
How much do you agree or disagree with the following statements?			
1. ASD has a genetic factor	3.31	0.61	0.52
2. ASD is caused by genes	2.85	0.66	0.67
3. ASD is associated with family history	2.92	0.63	0.68
4. ASD is an inherited disorder	2.75	0.68	0.70
Scale (total score)	11.82	2.07	
Internal Consistency (Cronbach’s α)	0.82		
Perceived Severity of ASD Scale (6 items)			
Response Scale: (1) Strongly Disagree, (2) Disagree, (3) Agree, (4) Strongly Agree			
How much do you agree or disagree with the following statements?			
1. ASD is a severe disorder	3.00	0.77	0.53
2. Individuals with ASD have problems living independently	3.04	0.70	0.63
3. The public discriminates against individuals with ASD	3.19	0.66	0.36
4. Individuals with ASD have fewer job opportunities	3.46	0.63	0.49
5. Compared to a child without ASD, it’s hard to raise a child with ASD	3.51	0.64	0.48
6. Health problems associated with ASD are severe	2.50	0.76	0.42
Scale (total score)	18.69	2.76	
Internal Consistency (Cronbach’s α)	0.74		
Perceived Benefits of Genetic Testing for ASD Scale (6 items)			
Response Scale: (1) Strongly Disagree, (2) Disagree, (3) Agree, (4) Strongly Agree			
How much do you agree or disagree with the following statements?			
1. ASD genetic testing identifies the cause of children’s ASD	2.37	0.69	0.33
2. ASD genetic testing promotes early detection and intervention for children with ASD	2.93	0.63	0.65
3. ASD genetic testing helps develop treatment plans targeting ASD-associated medical conditions for affected children	2.89	0.62	0.65
4. ASD genetic testing helps children with ASD make informed family planning decisions	2.80	0.70	0.54
5. ASD genetic testing helps children with ASD get more social support	2.56	0.76	0.54
6. Taking children with ASD to undergo ASD genetic testing contributes to ASD research	3.35	0.54	0.25
Scale (total score)	16.90	2.63	
Internal Consistency (Cronbach’s α)	0.75		
Perceived Barriers in Pursuing Genetic Testing for Children with ASD Scale (9 items)			
Response Scale: (1) Strongly Disagree, (2) Disagree, (3) Agree, (4) Strongly Agree			
How much do you agree or disagree with the following statements?			
1. ASD is not caused by genes	2.03	0.62	0.07
2. ASD genetic testing cannot improve the current situation of my child(ren) diagnosed with ASD	2.53	0.81	0.26
3. My child(ren) has/have already been diagnosed with ASD, so there’s no need to undergo this testing	2.10	0.73	0.46
4. Taking my child(ren) with ASD to undergo ASD genetic testing contradicts with my religious or cultural beliefs.	1.34	0.49	0.29
5. ASD genetic testing does more harm than good	1.59	0.61	0.56
6. ASD genetic testing can cause family conflicts	2.06	0.79	0.42
7. The procedure of undergoing ASD genetic testing is uncomfortable for my child (i.e., drawing blood)	2.76	0.90	0.28
8. The results of ASD genetic testing can cause public discrimination against my child(ren) with ASD	2.02	0.78	0.52
9. The results of ASD genetic testing can put the health insurance status of my child(ren) with ASD in jeopardy	2.22	0.87	0.46
Scale (total score)	18.65	3.58	
Internal Consistency (Cronbach’s α)	0.69		
Attitudes Toward Genetic Testing for ASD Scale—Product of Belief (5 items) and Value (5 items)			
1. Taking a child with ASD to undergo ASD genetic testing is a good thing	8.71	3.33	0.78
2. Taking a child with ASD to undergo ASD genetic testing is beneficial	9.06	3.32	0.74
3. Taking a child with ASD to undergo ASD genetic testing is necessary	6.00	3.65	0.83
4. All children diagnosed with ASD should undergo ASD genetic testing	6.16	3.72	0.86
5. All children with ASD characteristics or traits should undergo ASD genetic testing	6.19	3.64	0.82
Scale (total score)	36.12	15.52	
Internal Consistency (Cronbach’s α)	0.93		
Subjective Norms of Genetic Testing for ASD Scale—Product of Normative Belief (9 items) and Motivation to Comply (9 items)			
1. Spouse	5.92	4.36	0.51
2. Your family members on your side	4.49	3.47	0.68
3. Your family members on your spouse’s side	3.12	2.60	0.61
4. Physicians	6.01	3.65	0.75
5. Health care professionals other than physicians (e.g., nurses, social workers, occupational/physical/speech therapists, psychologists)	5.82	3.72	0.74
6. School teachers	3.94	3.34	0.71
7. Your friends	3.31	2.76	0.72
8. Other parents of children with ASD	5.13	3.70	0.61
9. General public	2.79	2.29	0.62
Scale (total score)	40.54	22.14	
Internal Consistency (Cronbach’s α)	0.89		
Self-efficacy in Pursuing Genetic Testing for Children with ASD Scale (6 items)			
Response Scale: From 0 (I am not confident at all) to 10 (I am 100% confident)			
How confident are you…			
1. in finding a time to take your child(ren) with ASD for ASD genetic testing?	6.34	3.08	0.50
2. that you are able to afford to take your child(ren) with ASD for ASD genetic testing?	3.57	3.18	0.54
3. in finding a suitable hospital or doctor to take your child(ren) with ASD for ASD genetic testing?	4.94	3.13	0.72
4. that you can make an appointment with an ASD genetic testing provider to take your child(ren) with ASD for ASD genetic testing?	4.76	3.12	0.77
5. that you can figure out the health insurance for ASD genetic testing	3.72	3.22	0.69
6. that your family members will support you in taking your child(ren) with ASD to undergo genetic testing for ASD?	6.57	2.95	0.49
Scale (total score)	29.91	13.91	
Internal consistency (Cronbach’s α)	0.84		
Intention in Pursuing Genetic Testing for Children with ASD Scale (6 items)			
Response Scale: (1) Extremely unlikely, (2) Unlikely, (3) Likely, (4) Extremely likely			
How likely are you to…			
1. organize your time to take your child(ren) with ASD to undergo ASD genetic testing?	2.67	0.92	0.83
2. pay out-of-pocket for ASD genetic testing for your child(ren) with ASD?	1.80	0.83	0.54
3. make an appointment with an ASD genetic testing provider to take your child(ren) with ASD for ASD genetic testing?	2.36	0.86	0.81
4. contact the health insurance company about the cost of ASD genetic testing for your child(ren) with ASD?	2.39	0.95	0.75
5. obtain your family members’ support to take your child(ren) with ASD to undergo ASD genetic testing?	2.28	0.93	0.58
6. take your child(ren) with ASD to undergo ASD genetic testing?	2.44	0.88	0.85
Scale (total score)	13.93	4.39	
Internal Consistency (Cronbach’s α)	0.90		

N = 308. α = alpha; POAGTS, Perceptions of ASD Genetic Testing Survey; ASD, Autism Spectrum Disorder.

**Table 2:** Factor loadings for exploratory factor analysis.

Item	Factor 1	Factor 2
Perceived Genetic Cause of ASD Scale (4 items)		
1. ASD has a genetic factor	0.70	
2. ASD is caused by genes	0.82	
3. ASD is associated with family history	0.84	
4. ASD is an inherited disorder	0.85	
Total variance explained = 64.80%	64.80%	
Perceived Severity of ASD Scale (6 items)		
1. ASD is a severe disorder	0.71	
2. Individuals with ASD have problems living independently	0.80	
3. The public discriminates against individuals with ASD	0.53	
4. Individuals with ASD have fewer job opportunities	0.68	
5. Compared to a child without ASD, it’s hard to raise a child with ASD	0.66	
6. Health problems associated with ASD are severe	0.60	
Total variance explained = 44.43%	44.43%	
Perceived Benefits of Genetic Testing for ASD Scale (6 items)		
1. ASD genetic testing identifies the cause of children’s ASD	0.48	
2. ASD genetic testing promotes early detection and intervention for children with ASD	0.82	
3. ASD genetic testing helps develop treatment plans targeting ASD-associated medical conditions for affected children	0.83	
4. ASD genetic testing helps children with ASD make informed family planning decisions	0.73	
5. ASD genetic testing helps children with ASD get more social support	0.71	
6. Taking children with ASD to undergo ASD genetic testing contributes to ASD research	0.39	
Total variance explained = 46.14%	46.14%	
Perceived Barriers in Pursuing Genetic Testing for Children with ASD Scale (9 items)		
1. ASD is not caused by genes	-	-
2. ASD genetic testing cannot improve the current situation of my child(ren) diagnosed with ASD	-	0.93
3. My child(ren) has/have already been diagnosed with ASD, so there’s no need to undergo this testing	-	0.81
4. Taking my child(ren) with ASD to undergo ASD genetic testing contradicts with my religious or cultural beliefs.	0.57	-
5. ASD genetic testing does more harm than good	0.69	-
6. ASD genetic testing can cause family conflicts	0.73	-
7. The procedure of undergoing ASD genetic testing is uncomfortable for my child (i.e., drawing blood)	0.48	-
8. The results of ASD genetic testing can cause public discrimination against my child(ren) with ASD	0.76	-
9. The results of ASD genetic testing can put the health insurance status of my child(ren) with ASD in jeopardy	0.65	-
Total variance explained = 47.29%	31.16%	16.13%
Attitudes Toward Genetic Testing for ASD Scale (5 items)		
1. Taking a child with ASD to undergo ASD genetic testing is a good thing	0.86	
2. Taking a child with ASD to undergo ASD genetic testing is beneficial	0.83	
3. Taking a child with ASD to undergo ASD genetic testing is necessary	0.89	
4. All children diagnosed with ASD should undergo ASD genetic testing	0.92	
5. All children with ASD characteristics or traits should undergo ASD genetic testing	0.89	
Total variance explained = 77.12%	77.12%	
Subjective Norms of Genetic Testing for ASD Scale—(9 items)		
1. Spouse	0.60	
2. My family members on my side	0.76	
3. My family members on my spouse’s side	0.69	
4. Physicians	0.81	
5. Health care professionals other than physicians (e.g., nurses, social workers, occupational/physical/speech therapists, psychologists)	0.81	
6. School teachers	0.78	
7. My friends	0.80	
8. Other parents of children with ASD	0.71	
9. General public	0.71	
Total variance explained = 55.37%	55.37%	
Self-efficacy Scale in Pursuing Genetic Testing for Children with ASD (6 items)		
1. in finding a time to take your child(ren) with ASD for ASD genetic testing?	0.63	
2. that you are able to afford to take your child(ren) with ASD for ASD genetic testing?	0.69	
3. in finding a suitable hospital or doctor to take your child(ren) with ASD for ASD genetic testing?	0.84	
4. that you can make an appointment with an ASD genetic testing provider to take your child(ren) with ASD for ASD genetic testing?	0.87	
5. that you can figure out the health insurance for ASD genetic testing	0.82	
6. that your family members will support you in taking your child(ren) with ASD to undergo genetic testing for ASD?	0.62	
Total variance explained = 56.06%	56.06%	
Intention in Pursuing Genetic Testing for Children with ASD Scale (6 items)		
1. organize your time to take your child(ren) with ASD to undergo ASD genetic testing?	0.90	
2. pay out-of-pocket for ASD genetic testing for your child(ren) with ASD?	0.66	
3. make an appointment with an ASD genetic testing provider to take your child(ren) with ASD for ASD genetic testing?	0.89	
4. contact the health insurance company about the cost of ASD genetic testing for your child(ren) with ASD?	0.83	
5. obtain your family members’ support to take your child(ren) with ASD to undergo ASD genetic testing?	0.69	
6. take your child(ren) with ASD to undergo ASD genetic testing?	0.91	
Total variance explained = 67.19%	67.19%	

N = 308 ASD, Autism Spectrum Disorder.

## Data Availability

The data presented in this study are available on request from the corresponding author, subject to any restrictions from the Institutional Review Board at the time of request.
